# Neuropathological Characterization of Dolphin Morbillivirus Infection in Cetaceans Stranded in Italy

**DOI:** 10.3390/ani12040452

**Published:** 2022-02-12

**Authors:** Federica Giorda, Paola Crociara, Barbara Iulini, Paola Gazzuola, Alessandra Favole, Maria Goria, Laura Serracca, Alessandro Dondo, Maria Ines Crescio, Tania Audino, Simone Peletto, Cristina Esmeralda Di Francesco, Maria Caramelli, Eva Sierra, Fabio Di Nocera, Giuseppe Lucifora, Antonio Petrella, Roberto Puleio, Sandro Mazzariol, Giovanni Di Guardo, Cristina Casalone, Carla Grattarola

**Affiliations:** 1 Istituto Zooprofilattico Sperimentale del Piemonte, Liguria e Valle d’Aosta, 10154 Torino, Italy; paola.crociara@gmail.com (P.C.); barbara.iulini@izsto.it (B.I.); paola.gazzuola@izsto.it (P.G.); alessandra.favole@izsto.it (A.F.); maria.goria@izsto.it (M.G.); laura.serracca@izsto.it (L.S.); alessandro.dondo@izsto.it (A.D.); mariaines.crescio@izsto.it (M.I.C.); tania.audino@izsto.it (T.A.); simone.peletto@izsto.it (S.P.); maria.caramelli@izsto.it (M.C.); cristina.casalone@izsto.it (C.C.); carla.grattarola@izsto.it (C.G.); 2 Institute for Animal Health and Food Safety (IUSA), Faculty of Veterinary Medicine, University of Las Palmas de Gran Canaria, Las Palmas de Gran Canaria, 35416 Canary Islands, Spain; eva.sierra@ulpgc.es; 3 Department of Prevention, Local Veterinary Services (ASLTO4), SS Sanità Animale, Piazza Gino Viano Bellandi, Cuorgnè, 10082 Torino, Italy; 4 Faculty of Veterinary Medicine, University of Teramo, Strada Provinciale 18 Località Piano d’Accio, 64100 Teramo, Italy; cedifrancesco@unite.it; 5 Istituto Zooprofilattico Sperimentale del Mezzogiorno, Via della Salute, 2, Portici, 80055 Napoli, Italy; fabio.dinocera@izsmportici.it (F.D.N.); giuseppe.lucifora@izsmportici.it (G.L.); 6 Istituto Zooprofilattico Sperimentale della Puglia e della Basilicata, Via Manfredonia 20, 71121 Foggia, Italy; antonio.petrella@izspb.it; 7 Istituto Zooprofilattico Sperimentale della Sicilia, Via Gino Marinuzzi, 3, 90129 Palermo, Italy; roberto.puleio@izssicilia.it; 8 Department of Comparative Biomedicine and Food Science, University of Padua, Legnaro, 35020 Padua, Italy; sandro.mazzariol@unipd.it; 9 Retired Professor of General Pathology and Veterinary Pathophysiology, Veterinary Medical Faculty, University of Teramo, Localita’ Piano d’Accio, 64100 Teramo, Italy; gdiguardo@unite.it

**Keywords:** cetacean morbillivirus, cetaceans, meningoencephalitis, demyelination, neuropathology, immunofluorescence

## Abstract

**Simple Summary:**

There is abundant literature reporting demyelination in dogs and pinnipeds affected by morbillivirus infection, but myelinopathy is poorly investigated in stranded cetaceans affected with the virus. Also, the neuropathogenesis of cetacean morbillivirus infection has not been fully clarified, leaving questions on cell tropism unanswered. A novel dolphin morbillivirus lineage of Atlantic origin circulating in Italian waters replaced the previous Mediterranean strain in late 2015; however, differences in virulence and pathogenesis between the two strains have not yet been documented. The aims of the present study were to: describe histopathological changes and immunohistochemical findings in the central nervous system of 31 cetaceans which tested positive on molecular investigations for the two dolphin morbillivirus strains; characterize by double indirect immunofluorescence staining the areas of myelin damage. The most frequently observed morbillivirus-associated lesions were astro-microgliosis, neuronal necrosis, spongiosis, malacia, and non-suppurative meningoencephalitis. Demyelination was detected by means of a specific myelin biomarker. Inside and around the demyelinated areas there were morbillivirus antigen-bearing cells of mainly neuronal and microglial origin, associated with marked astro and microglia reactivity. Molecular and immunohistochemical analysis suggested a higher neurotropic affinity of the novel circulating strain.

**Abstract:**

Cetacean morbillivirus (CeMV) is responsible for epidemic and endemic fatalities in free-ranging cetaceans. Neuro-inflammation sustained by CeMV is a leading cause of death in stranded cetaceans. A novel dolphin morbillivirus (DMV) strain of Atlantic origin circulating in Italian waters since early 2016 has caused acute/subacute lesions associated with positive immunolabelling of the virus. To date, myelin damage has not been fully documented and investigated in cetaceans. This study describes neuropathological findings in the brain tissue of 31 cetaceans found stranded along the Italian coastline and positive for DMV infection on molecular testing. Cell changes in the areas of myelinopathy were revealed by double indirect immunofluorescence. The most frequent DMV-associated lesions were astro-microgliosis, neuronal necrosis, spongiosis, malacia, and non-suppurative meningoencephalitis. Myelin reduction and areas of demyelination were revealed by means of a specific myelin biomarker. Morbilliviral antigen immunolabelling was mainly observed in neurons and microglial cells, in association with a marked activation of microglia and astrocytes. These findings extend our knowledge of DMV-associated brain lesions and shed light on their pathogenesis.

## 1. Introduction

Neuro-inflammation is a leading cause of death in stranded cetaceans, in which the brain is found to be the only organ affected in some cases [1,2,3]. Viral meningoencephalitis sustained by cetacean morbillivirus (CeMV) is the most frequent form of meningoencephalitis in cetaceans [2,3]. A member of the genus *Morbillivirus* (family Paramyxoviridae, subfamily Orthoparamyxovirinae), CeMV is the natural agent with the greatest impact on cetacean health and conservation worldwide [4]. This non-segmented single-stranded RNA virus includes three well-characterized strains: dolphin morbillivirus (DMV), porpoise morbillivirus, and pilot whale morbillivirus, plus four more strains recently identified in Hawaii and the southern hemisphere [5,6,7,8].

Genomic studies on DMV sequences circulating in the Mediterranean basin in the last 30 years show that the strain is generally well conserved [9,10]. In the last 6 years, however, the DMV Mediterranean strain has been substituted by a new variant called DMV northeast (NE)-Atlantic strain originating from the coasts of Galicia and Portugal [10,11,12]. No difference in virulence and disease severity between the two strains has been confirmed to date [4].

Neuropathological changes due to morbillivirus infection include demyelination, which is largely reported in both marine and terrestrial species and is commonly associated with canine and phocine distemper virus infection [13,14,15,16]. Few studies have described myelin changes in cetaceans with CeMV infection to date [12,17,18,19]. In some cases, infection has been confirmed by Luxol Fast Blue staining, which has not always proven effective in showing demyelination, however [20].

With the present study, we describe the histopathological changes in the central nervous system (CNS) of cetaceans with DMV infection found stranded along the Italian coastline in the period 2008–2020. In addition, we document differences in the virulence of the two strains circulating in the Mediterranean Sea.

To do this, we performed neuropathological characterization of areas of demyelination/hypomyelination by means of double indirect immunofluorescence (IF) staining to reveal cell changes and viral colonization of the neuronal and glial cell populations. Myelinopathy was confirmed by successful IF staining. To the best of our knowledge, this is the first study on neuropathological characterization by means of double IF staining in marine mammals with systemic CeMV infection. In addition, it is the first application of confocal laser-scanning microscopy for in-depth analysis of CeMV infection.

## 2. Materials and Methods

### 2.1. Materials

All animals were stranded cetaceans diagnosed during routine pathological analysis and cause-of-death assessment by the Italian cetacean stranding network, Istituti Zooprofilattici Sperimentali, veterinary public health institutions under the Italian Ministry of Health. The animals were examined and submitted to complete post mortem examination according to standard protocols [21].

Epidemiological (stranding location and date) and biological data (species, sex, age class, nutritional and decomposition status) were recorded. The animals were divided into three age categories (newborn-calf, juvenile-subadult, adult) based on total body length [21,22]. The decomposition condition of the carcasses (DCC) was classified as: code 1 (extremely fresh carcass, just died); code 2 (fresh); code 3 (moderate decomposition); code 4 (advanced decomposition); and code 5 (mummified or skeletal remains) [23]. The nutritional condition state (NCC) was classified as good, moderate or poor based morphologically on anatomical parameters (e.g., convexity of dorsal profile, rib prominence, amount of body fat).

During necropsy, tissue samples from major organs were collected and divided into three aliquots for subsequent analysis: one was kept frozen at −20 °C for microbiological analysis, one at −80 °C for biomolecular analysis, and the third was preserved for 10 to 14 days in neutral buffered formalin for histological and immunohistochemical (IHC) analysis. When available, ten areas of the CNS were sampled and examined: basal nuclei, thalamus, mesencephalon, pons, obex, spinal cord, and frontal, parietal, occipital, and cerebellar cortex. After fixing in 10% neutral buffered formalin, the tissue samples were embedded in paraffin, cut to 4 ± 2 μm thick, stained with haematoxylin and eosin (H&E), and examined under a light microscope.

Serological testing to screen for specific antibodies against morbillivirus and *T. gondii* was performed on serum, cerebrospinal fluid (CSF), and aqueous humour kept frozen at −20 °C, when available. The samples were tested by rapid serum agglutination (Rose Bengal plate test, RBT) using RBT antigen produced from *B. abortus* strain S99 [24,25] to detect anti-smooth *Brucella* spp. antibodies.

### 2.2. Neuropathological Investigation

The neuropathological reports of 188 brain samples submitted to the Italian National Reference Center for Diagnostic Activities in Stranded Marine Mammals (Centro di Referenza Nazionale per le Indagini Diagnostiche sui Mammiferi marini spiaggiati, C.Re.Di.Ma.) in the period 2007–2020 (69/188 in 2007–2015 and 119/188 in 2016–2020) were retrieved and further analysed for biomolecular investigation of DMV on the samples.

Morbillivirus IHC was carried out on tissue sections from all 188 CNS samples by means of a monoclonal anti-canine distemper virus (CDV) antibody (VMRD, Pullman, WA, USA) [25]. *T. gondii* IHC was carried out by means of a polyclonal serum of caprine origin (VMRD) on the tissue samples that showed microscopic and/or molecular evidence of protozoan infection [25].

In all, 31 animals had a morphological diagnosis of CNS inflammation or showed neurodegenerative and reactive changes referable to morbillivirus infection; 37 tested positive for DMV on PCR assay (9/37 were positive for the DMV Mediterranean variant and 28/37 for the DMV NE-Atlantic variant) [10]. Six animals were excluded: 3 were not evaluable due to advanced post mortem autolysis and 3 others presented no evidence of microscopic lesions. Only 9/66 animals (13%) resulted positive on molecular testing for DMV (Mediterranean variant) between 2007 and 2015, whereas 28/119 (23%) tested positive for DMV (NE-Atlantic variant) between 2016 and 2020.

Necropsy reports, including biological and epidemiological data, photographic material, and results of ancillary investigations of the 31 animals were retrieved and further analysed for assessment of the cases and determination of co-infection with other bacterial or viral neurotropic agents. A few cases were also previously published [1,10,12,26,27,28,29,30].

For the present study, the cerebral and the cerebellar cortex (because always present in the sample set) were re-examined according to the scheme devised by Sierra and colleagues (2020) [2] and slightly modified according to the description of CNS lesions reported in dogs affected by CDV [14,31]. Two independent pathologists scored the severity of meningitis, perivascular cuffing, astro-microgliosis, malacia, neuronal necrosis, and spongiosis as absent (–), minimal (+), mild (++), moderate (+ + +), and severe (+ + + +); intranuclear and/or intracytoplasmic inclusion bodies (INCIBs), haemorrhage, and IHC labelling for morbillivirus were scored as absent (–) or present (+). The stage of infection, termed acute (A), subacute (S) or chronic (C), was determined by multiparametric assessment of the type of neuropathological changes, antigen detection, and presence of co-infection [2,20,31,32]. Lesions of other anatomical regions and non-CNS lesions were recorded when present.

Three animals classified as DCC 2 (fresh carcass, well-preserved tissues), without co-infection with other bacterial and viral neurotropic pathogens, but presenting severe myelinopathy (ID 9, ID 10, ID 28) and brain immunolabelling for CDV were selected for further analysis by double staining indirect immunofluorescence (IF). The aim was to reveal myelin changes and characterize the brain cell populations targeted by the virus involved in demyelinated/hypomyelinated areas in DMV infection.

To do this, selected formalin-fixed paraffin-embedded (FFPE) tissues 4 ± 2 μm thick (cerebral cortex for ID 9; cerebellar cortex for ID 10; cerebral and cerebellar cortex for ID 28) characterized by severe spongiosis in white matter were processed for IF analysis. Negative control tissues consisting of FFPE cerebral and cerebellar cortex samples from a striped dolphin in DCC 2 tested negative on analysis (molecular, IHC, microbiological) for other neurotropic agents, e.g., DMV, herpesvirus (HV), *T. gondii*, and *Brucella* spp., without evidence of neuropathological changes.

In detail, antigen retrieval was performed using 10 mM citrate buffer (pH 6.1) at 95 °C for 20 min. Sections were incubated in blocking buffer (5% normal donkey serum, 0.3% Triton X-100 in 0.01 M PBS, pH 7.4) for 1 h at room temperature, then incubated for 24–48 h at 4 °C in a solution of 0.01 M PBS, pH 7.4, containing 0.1% Triton X-100, 2% normal donkey serum, and the primary antibodies. Commercially available primary antibodies (Abs) were used: murine monoclonal anti-CDV (1:500, VMRD Inc, Pullman, WA, USA), rabbit polyclonal (poAb) anti-GFAP (1:1000; Millipore, Burlington, MA, USA) (astrocytic marker), rabbit poAb anti-Iba 1 (1:1000, Wako Chemicals Corp., Osaka, Japan) (microglia marker), rabbit poAb anti-oligodendrocyte transcription factor (Olig2) (1:250, Millipore) (oligodendrocyte marker), rabbit poAb anti-myelin proteolipid protein (PLP) (1:500, Abcam, Cambridge, MA, USA) (myelin marker), and a rabbit poAb anti-NeuN (1:1000 Abcam) (neuron marker). After several washes, the sections were incubated with appropriate solutions of donkey Alexa 488 or Alexa 555 conjugated secondary antibodies (1:1000, Thermo Fisher Scientific, Waltham, MA, USA). The slides were then washed in PBS, counterstained with 4,6-diamidino-2-phenylindole (DAPI, 1:1000, KPL, Gaithersburg, MD, USA) and mounted with Fluoromount G (SouthernBiotech, Birmingham, AL, USA). As negative internal controls, primary antibodies were eliminated and replaced by nonimmune homologous serum. All fluorescence images were captured on a confocal laser scanning microscope (Leica TCS SP8, Leica Microsystem, Wetzlar, Germany).

Since no results were obtained with the Ab anti-Olig2 in IF (see Results) dual IHC was attempted for simultaneous localization of CDV and Oligo2. Antigen retrieval was obtained using 10 mM citrate buffer (pH 6.1) at 95 °C for 10 min. The sections were immersed in 3% hydrogen peroxide solution in methanol for 20 min to block endogenous peroxidases and subsequently pretreated with 3% normal horse serum in Tris-buffered saline with 0.1% Tween 20 detergent (TBST) for 20 min. CDV immunohistochemistry was performed first using anti-CDV Ab (VMRD Inc, Pullman, WA, USA) diluted 1:500 in TBST at 4 °C overnight. The slides were treated with appropriate secondary antibody conjugated to biotin, then developed using avidin-conjugated horseradish peroxidase (VECTASTAIN^®^ ABC-HRP Kit, Peroxidase-Mouse IgG, PK4002, Vector Laboratories, Burlingame, CA, USA) with DAB (K3467, Agilent Dako, Santa Clara, CA, USA) as substrate. The sections were then submitted to Oligo2 immunohistochemistry for testing the two markers: the rabbit poAb anti-Olig2 (1:250 Millipore, Burlington, MA, USA) and the rabbit monoclonal anti-Olig2 Ab (1:100 Abcam, Cambridge, MA, USA) diluted in TBST and incubated at 4 °C overnight. Both anti-Oligo2 antibodies were revealed using the VECTASTAIN^®^ ABC-HRP Kit, Peroxidase -Rabbit IgG (PK4001, Vector Laboratories, Burlingame, CA, USA) with peroxidase substrate VIP (SK-4600, Vector Laboratories, Burlingame, CA, USA). Table 1 presents the abs and their characteristics.

Finally, anti-myelin, anti-GFAP, anti-NeuN, and anti-Iba1 abs were validated by means of Western blot assay for their use in striped dolphin brain samples (Supplementary Materials Figure S1).

### 2.3. PCR and Sequence Analysis in CNS

Molecular detection of dolphin morbillivirus (DMV) was achieved from a fresh-frozen sample of CNS (approximately 1 g) consisting of several subsamples from different anatomical areas of the brain and the cerebellum. This was done to increase diagnostic sensitivity—given the multifocal localization of the infection [32]—with an end-point RT-PCR using degenerate primers to amplify 287 bp of the nucleoprotein (N) gene [26].

All CNS samples (*n* = 31) were screened for other common neurotropic pathogens for cetaceans [1,2], including herpesvirus, *T. gondii*, and *Brucella* spp. In detail, herpesvirus detection was performed with a nested PCR using degenerate primers to amplify a region of the DNA polymerase gene [33]. *T. gondii* DNA was detected with a nested-PCR targeting the ITS1 region [34], and *Brucella* spp. with a TaqMan® Brucella species detection kit (Applied Biosystems, Foster City, CA, USA) for real-time PCR targeting the IS711 gene.

For DNA and RNA extraction, tissue samples (30–50 mg) were physically disrupted using a TissueLyser II homogenizer (Qiagen, Hilden, Germany) by high-speed shaking in plastic tubes with stainless-steel beads (5 mm diameter). Genomic DNA was then extracted from the disrupted tissues with an All-Prep DNA/RNA Mini kit (Qiagen, Hilden, Germany) according to the manufacturer’s instructions.

The PCR products were analysed by electrophoresis on 2% agarose gel containing GelRed (Biotium, Fremont, CA, USA), compared with molecular weight markers, and then photographed on a Gel-Doc UV transilluminator system (Bio-Rad, Hercules, CA, USA).

For the DMV and herpesvirus assays, the amplicons were directly sequenced using PCR primers on a 3130XL Genetic Analyzer (Thermo Fisher Scientific Inc., Waltham, Massachusetts, USA). The sequences were aligned using SeqMan software (Lasergene package. DNASTAR Inc., Madison, WI, USA) to obtain a consensus sequence and compared with available sequences retrieved from the National Center for Biotechnology Information (NCBI) database with the BLAST tool (Available online: http://blast.ncbi.nlm.nih.gov/Blast.cgi; accessed on 10 November 2021). Molecular characterization to discriminate between the DMV strains was carried out as described by Bellière and colleagues [35].

### 2.4. Microbiological Analysis of the CNS: Standard and Specific for Brucella Isolation

All CNS samples (*n* = 31) were processed for standard aerobic, anaerobic, and microaerobic (5% CO_2_) bacterial culture and identification by biochemical and/or molecular analysis. Cultures for *Brucella* spp. were performed following international recommendations [36] and using selective and non-selective solid media and enrichment broths to enhance the chance of isolation.

### 2.5. Statistical Analysis

Statistical analyses were performed using STATA 17.1 (StataCorp College Station, Texas, USA). As most of the variables are ordinal, we performed univariate analysis with the non-parametric Wilcoxon–Mann–Whitney test to compare either the individual variable (sex, age class, species) or the score of the CNS lesions (meningitis, perivascular cuffing, astro-microgliosis, malacia, neuronal necrosis, spongiosis, intranuclear and/or intracytoplasmic inclusion bodies (INCIBs), haemorrhage, and stage of infection) or the molecular or the immunohistochemical findings between the two DMV strains. Statistical significance was set at *p* < 0.05. Multivariate analysis was performed by means of multi-level mixed-effect logistic models, including the strain as the dependent variable, age class, and CNS lesion score, the molecular and the immunohistochemical findings as independent variables, and the individual animal as a random effect.

## 3. Results

We analysed 31 cetaceans with a microscopic diagnosis of brain inflammation or showing neurodegenerative and reactive changes associated with molecular confirmation of DMV infection: 29 striped dolphins (*Stenella coeruleoalba* Meyen, 1833) (93.5%) and 2 bottlenose dolphins (*Tursiops truncatus* Montagu, 1821) (6.5%).

History and stranding data are presented in Supplementary Materials Table S1. Males (20/31; 64.5%) outnumbered females (11/31; 35.5%). The majority were juvenile-subadults (17/31; 54.8%) while the others were adults (14/31; 45.2%); no calves/newborns were present.

Only 3/31 carcasses (9.7%) were classified as very fresh (DCC 1), most were fresh (20/31; 64.5%) (DCC 2) and 8/31 (25.8%) were moderately decomposed (DDC 3). With regard to nutritional status, 9/31 (29%) showed good, 6/31 (19.3%) moderate, and 16/31 (51.6%) poor body condition.

**Figure 1:**
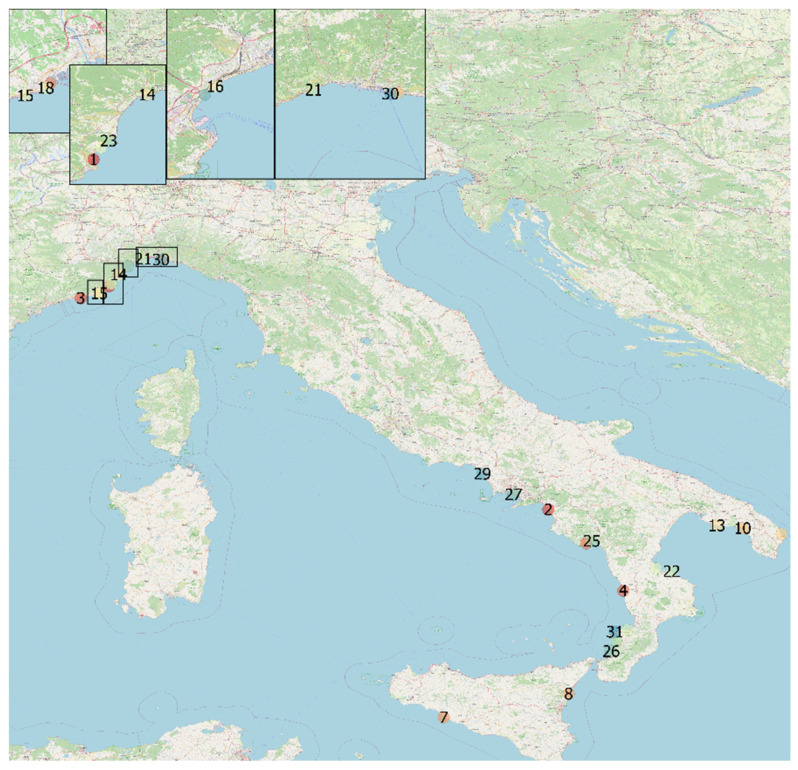
Stranding sites of cetaceans infected by dolphin morbillivirus, Italy, 2018–2020. Geographical mapping was created by M.I.C. with QGIS 3 software (QGIS Geographic Information System, QGIS association. www.QGIS.org, accessed on 29 January 2022) using the geographical coordinates found from the strandings.

The animals were found stranded over a 13-year period (from October 2008 to April 2020) along the Italian coastline [13/31 (41.9%) in Liguria, 7/31 (22.6%) in Calabria, 5/31 (16.1%) in Campania, 3/31 (9.7%) in Apulia, and 3/31 (9.7%) in Sicily]. A map indicating stranding date and location is shown in Figure 1.

Supplementary Materials Table S2 presents the results of molecular, immunohistochemical, microbiological, and serological analysis, along with the type of infection (systemic or localized) based on whether organs other than the CNS tested positive by molecular testing for DMV [32]. The animals found stranded between 2008 and 2015 were diagnosed with the Mediterranean DMV variant, whereas those found between 2016 and 2020 were diagnosed with the DMV NE-Atlantic variant. Except for ID 2, in which the brain was the only organ that tested positive for DMV, all the other animals presented systemic DMV infection of at least two other organs besides the CNS and tested positive for the virus [32] (data not shown).

With regard to common neuropathogens in cetaceans (Sierra et al. 2020), 10/31 animals (32%) presented with cerebral co-infection: *T. gondii* (7/10) (ID 1, 5, 16, 24, 26, 29, 30), *T. gondii* and *Brucella ceti* (1/10) (ID 14), ˠHV (1/10) (ID 11) and *T. gondii* and ˠHV (1/10) (ID 20). Overall, immunostaining for *T. gondii* abs was positive in 6/9 animals. In addition, microbiological analysis revealed *P. damselae* subsp. *damselae* in 5 animals (ID 11, 14, 21, 30, 31), *Salmonella* 1,4,[5], 12:i: in ID 16, *L. ivanovii* in ID 10, and *L. innocua* in ID 21.

Overall, immunostaining against CDV abs was positive in 24/31 animals. Noteworthy with regard to the strains circulating in the Mediterranean, 3 of the animals were from the group of 6 (50%) that tested positive on molecular testing for the DMV Mediterranean variant, while 21 were from the group of 25 animals (80%) that tested positive for the DMV NE-Atlantic variant. Anti-*Morbillivirus,* anti*-T. gondii,* and anti*-Brucella* spp. abs were detected in 13 animals. Anti-morbillivirus antibodies were detected in the serum of ID 6 (1:40), ID 11 (1:40), ID 14 (1:8), ID 17 (>1:256) and in the aqueous humour of ID 28 (1:128). Anti-*T. gondii* abs were detected in ID 1 (1:80 serum), ID 3 (>1:160 serum), ID 6 (1:80 serum), ID 11 (>1:40 serum), ID 14 (>1:40 serum, CSF, aqueous humour), ID 15 (>1:40 serum), ID 16 (>1:640 serum and aqueous humour), and ID 30 (>1:40 serum and aqueous humour). No evidence of anti-*Brucella* spp. abs was found in sera, CSF or aqueous humour in the samples from either of the dolphins tested. No results were obtained from ID 21 because of haemolysis.

**Figure 2:**
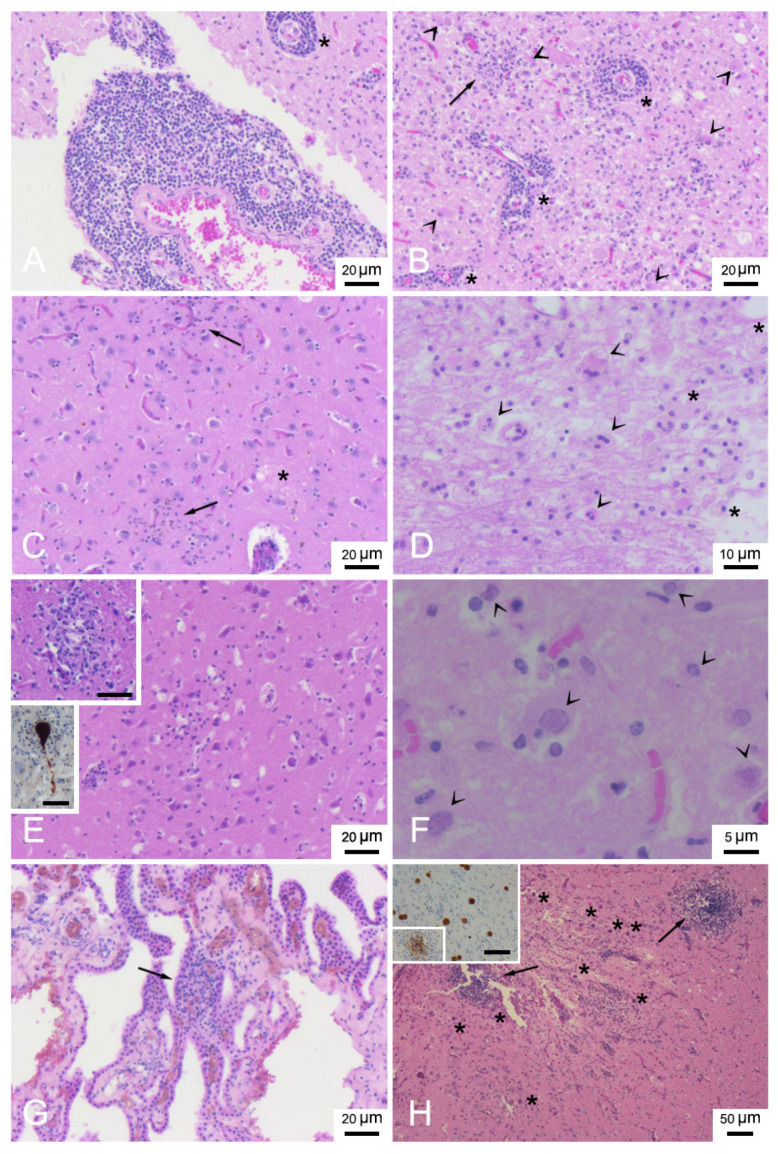
Neuropathologic lesions in stranded cetaceans with DMV infection. (**A**) Frontal cortex (ID 19). Severe non-suppurative meningitis and perivascular cuffing (asterisk). HE. (**B**) Frontal cortex (ID 19). Severe encephalitis with non-suppurative perivascular cuffing (asterisks), neuronophagic nodule of microglia cells (arrow) and diffuse astro-microgliosis with gemistiocytes (arrowheads). HE. (**C**) Frontal cortex (ID 16). Microgliosis (arrows) and oedema (asterisk), HE. (**D**) Cerebellum (ID 7). Malacic area with gitter cells (asterisks) and syncytia cells (arrowheads), HE. (**E**) Parietal cortex (ID 31). Diffuse acute neuronal necrosis (eosinophilic and shrunken neurons) and microgliosis. Upper inset: occipital cortex (ID 24). Neuronophagic nodule of microglia cells. Scale bar = 20µm. HE. Lower inset: pons (ID 16). Positive labelling of a neuron inside a neuronophagic nodule. Scale bar = 25 µm. IHC for DMV. (**F**) Occipital cortex (ID 19). Eosinophilic intranuclear inclusion bodies (arrowheads). HE. (**G**) Plexus choroideus (ID 14). Focal and minimal non-suppurative plexus choroiditis (arrow). HE. (**H**) Frontal cortex (ID 16). Severe necrotizing granulomatous encephalitis with protozoan cysts (asterisks) and two nodules of microglia and mononuclear inflammatory cells (arrow). HE. Inset: Frontal cortex (ID 16). Positive labelling of a nodule of microglia and mononuclear inflammatory cells and several protozoan cysts. Scale bar = 50 µm. IHC for *Toxoplasma gondii*.

**Figure 3:**
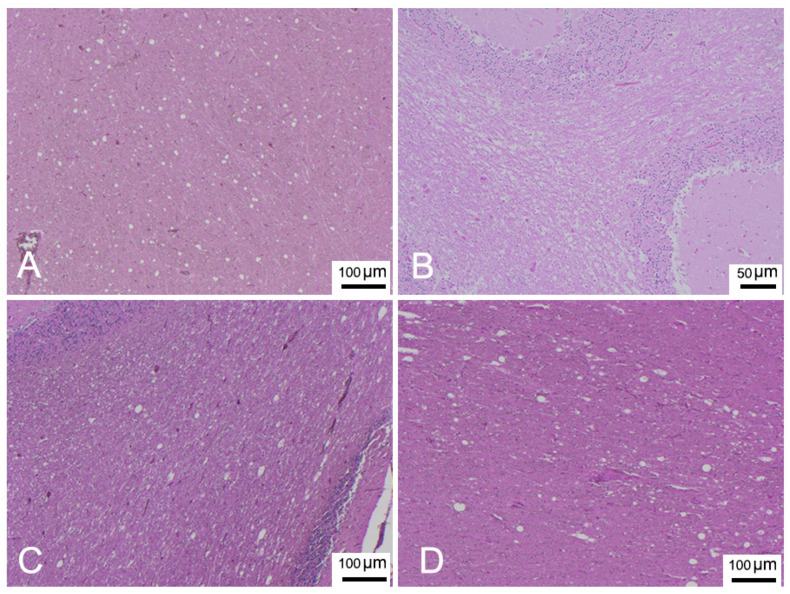
(**A**) Parietal cortex (ID 9). Spongiosis in white matter. HE. (**B**) Cerebellar cortex (ID 10). Severe myelinopathy, HE. (**C**) Occipital cortex (ID 28). Spongiosis in white matter. HE. (**D**) Cerebellar cortex (ID 28). Spongiosis in white matter. HE.

Table 2 presents a description of CNS DMV-associated lesions and includes inflammatory lesions due to non-suppurative meningoencephalitis, which were classified as subacute or chronic, and acute lesions due to encephalopathy/encephalitis, comprising mainly neurodegenerative and reactive changes with minimal or absent inflammatory components [20,31,32]. In detail, the chronic form was described in only 1 animal (ID 17). Subacute forms were assigned to 17 animals (ID 1, 2, 6, 12, 14, 16, 18-24, 26, 28, 29, 31) presenting meningitis (Figure 2A) and mild to moderate perivascular cuffing (Figure 2A,B) consisting of lymphocytes and plasma cells. Acute forms were found in 13 animals (ID 3-5, 7-11, 13, 15, 25, 27, 30). Minimal to severe astro-microgliosis (Figure 2B,C,E) was present in 24 animals, frequently in those presenting inflammatory forms. Mild to moderate malacia (Figure 2D) was detected in 14 animals presenting inflammatory patterns (severe malacia was observed in 2 animals with acute forms [ID 7 and 8]). Minimal to severe neuronal necrosis (Figure 2B,E) was observed in 23 animals. Mild to severe spongiosis in white matter was a common feature in 27 animals. Three showed severe myelinopathy (ID 9, 10, 28) (Figure 3) and were selected for characterization by double IF and IHC. INCBs were detected in only one animal (ID 19) (Figure 2G), which presented severe subacute meningoencephalitis. Finally, haemorrhage was present in 16 animals.

Lesions in other anatomical regions included: diffuse non-suppurative meningoencephalitis in all sections examined, along with the cerebellar and the cerebral cortex, in 8 animals (ID 1, 6, 14, 16, 17, 21, 23, 28), non-suppurative plexus choroiditis (Figure 2G) in 6 animals (ID 1, 4, 11, 14, 19, 24), non-suppurative myelitis in 2 animals (ID 3 and 17), oedema (Figure 2C) in 3 animals (ID 8, 16, 22). Associated lesions comprised syncytia (Figure 2D) in 11 animals (ID 1, 2, 7, 8, 14, 16, 19, 21, 23, 24, 27), protozoan tissue cysts associated with granulomatous encephalitis (Figure 2H) in 4 animals (ID 14, 16, 24, 26), protozoan tissue cysts alone in 1 animal (ID 1), Purkinje cell loss in 2 animals (ID 10 and 24), vasculitis in 2 animals (ID 19 and 21), suppurative and pyogranulomatous encephalitis in 1 animal each (ID 12 and 19, respectively). Three animals (ID 5, 20, 29) presented no protozoan cysts, although molecular analysis indicated co-infection with the parasite.

No differences in histopathological features and in stage of infection were found between the two strains (unfortunately, no cases from the 2013 unusual mortality event (UME) were included in the present study). No statistically significant difference between the two strains for any of the parameters was shown by either univariate or multivariate analysis.

**Figure 4:**
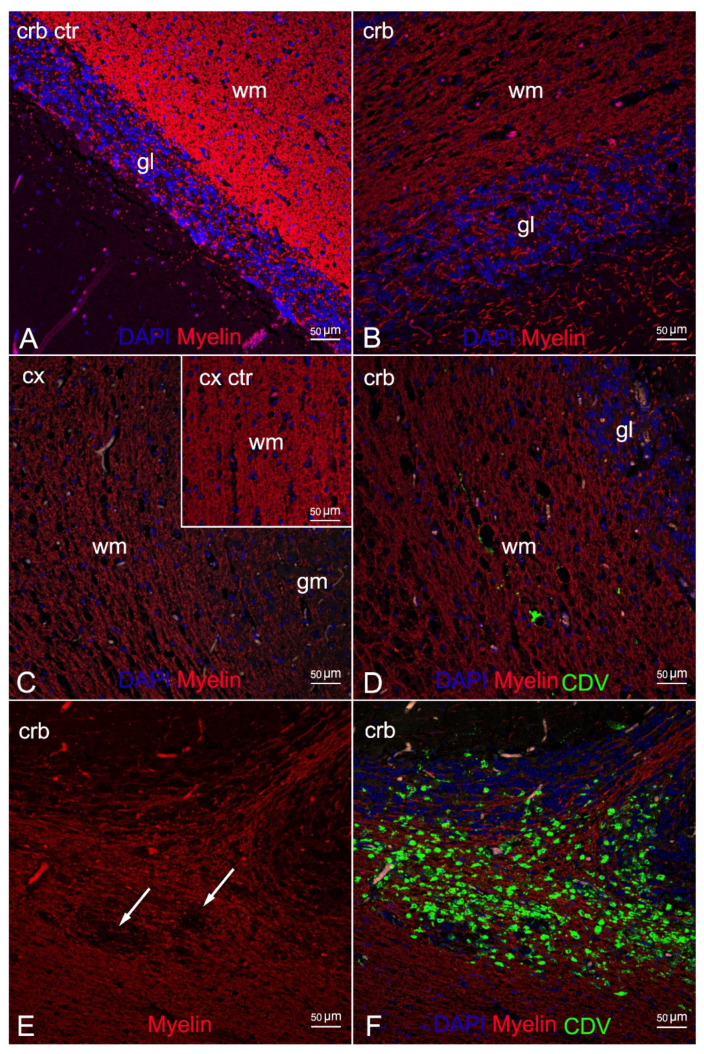
Morphological characterization of myelinopathy by means of double IF for myelin and DMV. (**A**) Cerebellar cortex (negative control). Myelinated white matter. (**B**) Cerebellar cortex (ID 28). Marked reduction in the myelin density with partial loss of tissue organization. (**C**) Cerebral cortex (ID 9). Severe reduction of myelin density, 20x. Inset: cortical cortex (negative control). Myelinated white matter. (**D**) Cerebellar cortex (ID 28). Limited DMV infection of the demyelinated white matter. (**E**) Cerebellar cortex (ID 10). Demyelinated white matter with complete loss of myelin (arrows). (**F**) Cerebellar cortex (ID 10). Severe DMV infection of the demyelinated white matter and the granular layer. Legend: wm, white matter; gm, grey matter; gl, granular layer; Cx, cortex; Crb, cerebellum; Ctr, negative control; DAPI, 4,6-diamidino-2-phenylindole; CDV, canine distemper virus.

IF labelling with anti-myelin ab in areas characterized by myelin damage in 3 animals (ID 9, 10, 28) revealed myelinopathy, with a marked reduction in myelin density and a partial loss of tissue organization (Figure 4B,C) compared to the negative control (Figure 4A), and large areas completely devoid of myelin (Figure 4E). In addition, double staining with CDV indicated viral infection in the demyelinated areas, more pronounced in ID 10 and less so in ID 28 (Figure 4D,F, respectively).

**Figure 5:**
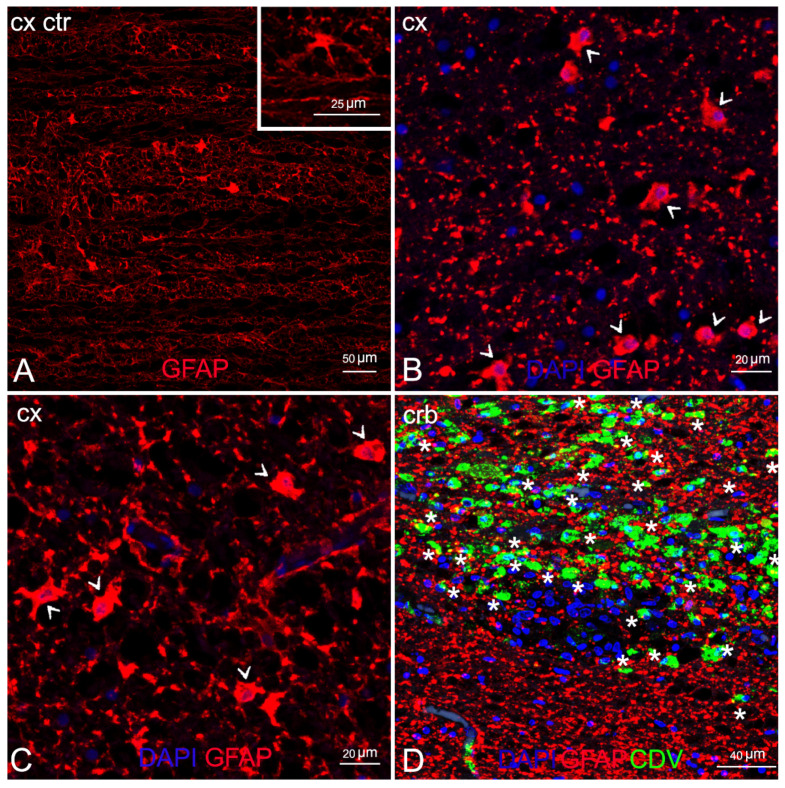
Characterization of demyelinated areas by means of double IF for astrocytes and DMV. (**A**) Cerebral cortex (negative control). Inset: cerebral cortex (negative control). Astrocyte in resting-state. (**B**) Cerebral cortex (ID 9). Several reactive astrocytes (arrowhead). (**C**) Cerebral cortex (ID 28). Several reactive astrocytes (arrowhead). (**D**) Cerebellar cortex (ID 10). Viral infection in astrocytes, co-localization anti-CDV and anti-GFAP visible in yellow (asterisks). Legend: Cx, cortex; Crb, cerebellum; Ctr, negative control; DAPI, 4,6-diamidino-2-phenylindole; CDV, canine distemper virus.

Comparison of double staining between the anti-GFAP and the anti-CDV abs showed marked astrocytosis, with reactive astrocytes inside and around the demyelinated areas (Figure 5B,C) but very little viral colonization in these cells, with a single area of overlapping in ID 10 (Figure 5D).

**Figure 6:**
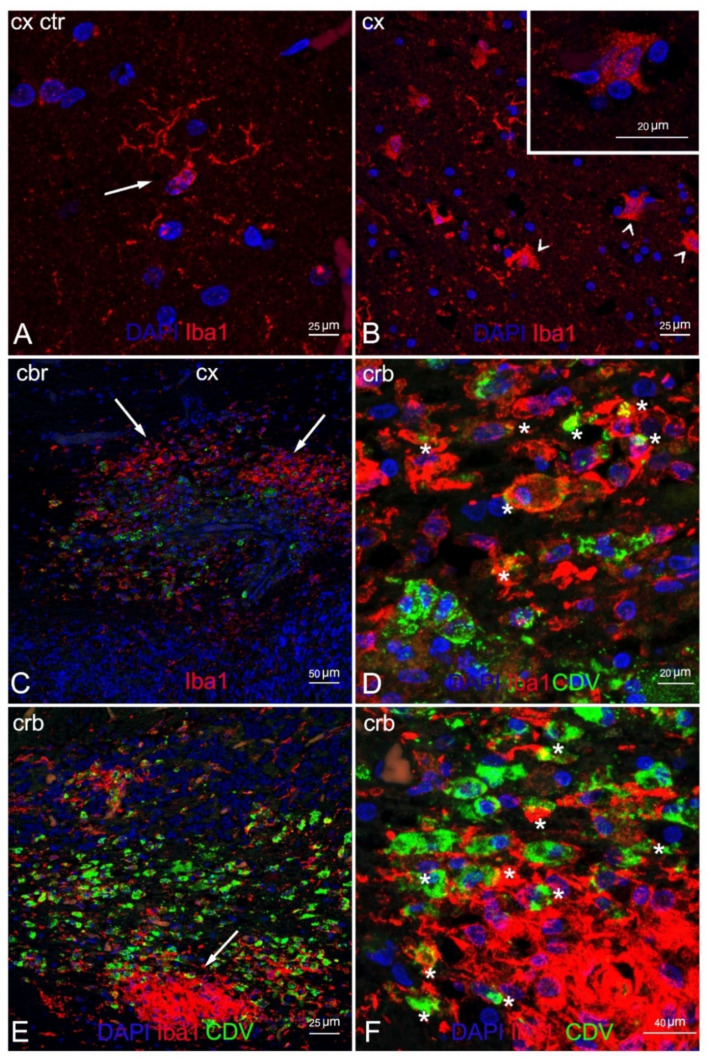
Characterization of demyelinated areas by means of double IF for microglia and DMV. (**A**) Cerebral cortex (negative control). Microglia in normal condition (resting state) (arrow). Note the typical ramified morphology characterizing these cells in healthy tissues. (**B**) Cerebral cortex (ID 28). Reactive microglia in demyelinated white matter (arrowhead). Inset: magnification of reactive microglia. (**C**) Cerebellar cortex (ID 10). Microgliosis in white matter (arrow) infected with DMV. (**D**) Magnification of image 4C. Marked co-localization of microglia and DMV (asterisks). (**E**) Cerebellar cortex (ID 10). Microglial nodule (arrow) positive for DMV. (**F**) Magnification of image 6E. Co-localization of microglia and DMV (asterisks) in the periphery of the nodule. Legend: Cx, cortex; Crb, cerebellum; Ctr, negative control; DAPI, 4,6-diamidino-2-phenylindole; CDV, canine distemper virus.

**Figure 7:**
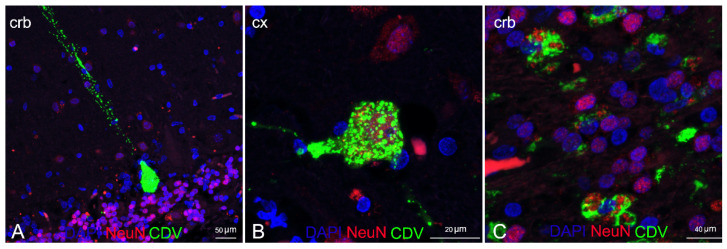
Characterization of DMV CNS infection by means of double IF for neurons and DMV. (**A**) Cerebellar cortex (ID 28) Purkenje cell positive for positive for DMV. (**B**) Cerebral cortex (ID 28). Cortical neuron positive for DMV. (**C**) Cerebellar cortex (ID 10). Neurons of granular layer positive for DMV. Legend: Cx, cortex; Crb, cerebellum; DAPI, 4,6-diamidino-2-phenylindole; CDV, canine distemper virus.

Double IF labelling with anti-CDV, anti-Iba1, and anti-NeuN demonstrated the neuronal and microglial origin of the morbillivirus antigen-bearing cells (Figure 6 and Figure 7). In addition, severe and diffuse microglia activation was observed in all tissue sections (Figure 6B). No oligodendrocyte origin of DMV infection could be found by either double IF or through double IHC, since both anti-Olig2 markers employed failed in staining the oligodendroglia cells.

## 4. Discussion

CeMV has been responsible for several outbreaks and inter-epidemic lethal diseases worldwide. Three unusual mortality events (UMEs) associated with DMV infection in the Mediterranean basin were reported for Italy and Spain (2011, 2013, 2016) [12,38,39], in addition to single disease descriptions of DMV infection along the Italian coastline reported by the C.Re.Di.Ma. in standard annual mortality rates [40,41,42,43,44].

Despite high levels of biomolecular positivity to DMV, the majority of the infected animals in the 2011 and 2013 Mediterranean UMEs presented milder lesions and pathological changes not directly referable to the virus but rather indicating chronic systemic DMV infection associated with secondary infection accompanied by scarce or absent immunoreactivity towards the virus [19,32,39]. In contrast, during the 2016 UME, pathological findings suggestive of acute/subacute disease were noted that resembled those reported during the Mediterranean outbreaks of 1990–1992 and 2006–2008 [45], with *Morbillivirus* immunostaining becoming a common feature in tissues testing positive on molecular tests [12].

Recent phylogenic and phylogeographic analysis [10] on 16 strains that circulated in the last 30 years in the Mediterranean confirmed a general well-conserved homology among the strains, with an overall sequence identity >98%. A novel lineage of Atlantic origin, named NE-Atlantic strain because first detected in animals found stranded along the coasts of Galicia and Portugal in 2011–2013, started to replace the Mediterranean strain by late 2015 in Italian waters [10,11,12]. The first description of the NE-Atlantic strain in the Mediterranean basin refers to a sperm whale (*Physeter macrocephalus* Linnaeus, 1758) found stranded on the coast of Vasto (CH) in 2014 [10] during a mass stranding episode that involved seven individuals [46]. The animal may have been responsible for the transmission of the new strain in Italian waters, given the role of spillover host of this species [47] and the date of stranding recorded almost 2 years before the first description in small odontocetes inhabiting the same geographical area.

For this study, we retrieved the neuropathological reports on brain tissue samples from 188 cetaceans submitted to the C.Re.Di.Ma. for 2007–2020. Between 2007 and 2015, during which the DMV Mediterranean strain began circulating, 13% (9/69) of the tissue samples tested positive on molecular testing. Differently, between 2016 and 2020, when only the NE-Atlantic strain was known to circulate in Italian waters, 23% (28/119) of the brain samples tested positive for the virus. With reference to antigen detection by IHC analysis, 50% (3/6) of the tissue samples obtained during the first reporting period (DMV Mediterranean strain) showed positive labelling, whereas the percentage of brains with positive immunolabelling was increased by 80% (21/25) for the second reporting period (DMV NE-Atlantic strain). Our data, albeit not statistically significant because of the small number of specimens infected with the DMV Mediterranean strain that precluded analysis, suggest a higher neurotropic potential of the NE-Atlantic strain compared to the Mediterranean strain. The new variant, because of its higher virulence for the CNS, does not allow the affected animals to survive the acute/subacute stage of infection, different from observations recorded during the UMEs of 2011 [19] and 2013 [39] in which the chronic forms predominated.

Almost half (45.2%) of the animals infected with the new variant were adults. This could mean that the virus was circulating within an immunologically naïve population (unfortunately, the serological analysis did not discriminate between the two variants), and resulted in severe disease. This observation differs from the events reported during the two UMEs which, probably due to a lack of specific antiviral immunity, involved mainly new-born, juveniles, and subadults [38,39].

Further studies would be of paramount importance to confirm our hypothesis for a higher neurotropic potential of the NE-Atlantic strain, with a focus on other target organs (e.g., lungs, spleen, lymph nodes) to better characterize systemic disease severity.

The present study describes the neuropathological findings in cetaceans found stranded along the Italian coastline and which received a morphological diagnosis of CNS inflammation or showed neurodegenerative and reactive changes referable to morbillivirus infection, as confirmed by molecular testing. The neuropathological changes due to DMV infection we noted are shared by previous reports [1,2,18,20,32] and are consistent with an earlier stage of acute and subacute disease: neurodegenerative and reactive alterations accompanied or not by mild-to-severe inflammatory lesions. In many animals (74%) there was minimal-to-severe diffuse neuronal necrosis in both the acute and the subacute/chronic forms, with shrunken eosinophilic neurons and neuronophagic nodules, respectively. This high percentage differs from a recent report by Sierra and colleagues [2] and may be attributable to the stage of infection (chronic forms) described in animals from the Canary Islands. Malacia was commonly observed in acute presentations characterized by gitter cells in almost half of the animals (45%). Syncytia, present in one-third of the animals in our sample, may be considered a characteristic feature of DMV since it was found in brains not co-infected with neurotropic agents (except for ID 1, 14, 16), as reported by Sierra and colleagues [2]. Half of the animals showed haemorrhage, a non-specific lesion observed also in cases of co-infection.

The most common CNS changes were astro-microgliosis and spongiosis of the white matter: astro-microgliosis was noted in 77% of animals, often in moderate and severe form, while spongiosis was seen in nearly all animals (87%) except for four (ID 21, 27, 29, 31). Minimal-to-severe non-suppurative inflammatory infiltrate in the meninges and around the vessels was responsible for a gradual transition to later stages of the disease. It was observed in almost 68% of animals (ID 5 and 9 presented acute infections). Only one animal (ID 19) presented a few neurons and astrocytes with eosinophilic intranuclear inclusion bodies, a typical and rather rare finding in DMV brain infection [2,20]. No co-infection with herpesvirus was reported in this animal. Purkinje cell loss was observed in 6% of the animals; this alteration is considered reliable only in very fresh and fresh cerebellum tissues (DDC 1 and 2). In line with previous data [2,20], vasculitis was seldom present (6%) and not associated with co-infection with common cetacean pathogens in our study. Mild-to-moderate non-suppurative plexus choroiditis was noted in six animals (19%), four of which with co-infection with other neurotropic agents. Of note is that this neuroanatomical region is the initial site of DMV brain invasion by the hematogenous route [48].

The spinal cord was rarely included in the sampling set during necropsy. Spinal cord lesions (non-suppurative myelitis) were observed in only two animals. In addition, diffuse non-suppurative meningoencephalitis was diagnosed in eight animals for which the whole cerebrum was available (exam not limited to only the cerebral and cerebellar cortex). These data highlight the importance of exhaustive sampling during post mortem examination.

In line with a previous description [20], immunostaining for the virus was not consistent for all tissue samples: positive labelling was achieved in 77% of cases (24/31).

We detected cerebral co-infection by common cetacean neurotropic agents [2] in one-third of the animals (10/31, 32%). The most frequent was *T. gondii* (9/10), alone (7/10) or associated with *Brucella ceti* or ˠHerpesvirus (HV) in the other two animals. Moreover, ˠHV was also detected in one supplementary case. The activity of these agents in the pathogenesis of lesions cannot be ruled out, since histopathological alterations in co-infection can be a consequence of the overlapping of two or more pathogens [2].

*T. gondii* is a zoonotic coccidian protozoan regarded as a primary pathogen for cetaceans and responsible for toxoplasmosis, a major emerging disease in these species [25,49]. Five out of nine infected animals (ID 1, 14, 16, 24, 26) showed tissues cysts, often associated with moderate-to-severe granulomatous encephalitis, as reported in terrestrial and marine species [2,50]. Tissue cysts and zoites were confirmed by IHC labelling. Three animals tested positive on molecular testing but no cysts could be found in the sections we examined.

Brucellosis is a widespread zoonosis of public health and economic concern in many areas of the world [28]. Meningoencephalomyelitis is one of the most common lesions caused by *B. ceti* in cetaceans, [2], although infections often show subclinical development in these species [51]. The only animal (ID 14) co-infected with *B. ceti* (and *T. gondii*) showed moderate non-suppurative meningitis and focal and minimal plexus choroiditis. Although both lesions are regarded as characteristic for this bacteria [2,28], these features have also been described in animals presenting with DMV infection alone and characterized by a more severe pattern (ID 19).

Herpesvirus infection carries neuropathological significance in cetaceans. CNS damage has been confirmed in association with αHV [52,53,54], and lesions typically referable to the infection are consistent with non-suppurative meningoencephalitis. Identifying the pathogenic role of ˠHV is more difficult, since the association between encephalitis and meningoencephalitis and ˠHV infection was first established in 2021 [55] in 8 animals and in one more with co-infection with αHV. Since the pathological changes observed in 2 animals with ˠHV co-infection (ID 11 and 20) closely matched the lesions characteristic for DMV, a specific role for ˠHV in the neuropathogenesis of these lesions cannot be confirmed or ruled out.

Noteworthy with regard to cerebral co-infection with bacterial agents other than *Brucella* spp. is the isolation of *Photobacterium damseale* subs. *damselae* in five animals, of *Salmonella* 1,4,[5],12:i:-, *L. ivanovii*, and *L. innocua* in 1 animal each. *P. damselae* subsp. *damselae* is increasingly found in stranded marine mammals but little is known about its precise etiological responsibility [39,56].

As concerns the pathological significance of *Salmonella* 1,4,[5],12:i:- in cetaceans, very few cases have been documented to date, one of which is ID 16 [27]. The detection of *Salmonella* 1,4,[5],12:i:- in the brain and other tissues and biological fluids, including the blood, was attributed to a septicemic form of infection, with evidence of microscopic intestinal and vascular lesions (intestinal necrosis, vascular embolus in intestinal mesentery) [27].

*Listeria ivanovii*, widely distributed in nature, is a potential cause of listeriosis in ruminants and in immunocompromised humans, which is why it is recognized as a pathogenic agent along with *Listeria monocytogenes* [57], although a neuropathological role has not yet been confirmed [57,58]. The lesions observed in the brain of ID 10 were clearly suggestive of viral infection; a role of the bacteria in the neuropathogenesis of these features was ruled out for this reason.

*L. innocua* is generally considered nonpathogenic. Although two cases of *Listeria innocua* meningoencephalitis were described in domestic animals, one in a ewe (*Ovis aries* Linnaeus, 1758) [59] and the other in a bull (*Bos taurus* Linnaeus, 1758) in northwest Italy [60], no descriptions in marine mammals have been reported to date. Histopathological findings observed in the fatal case involving the bull [60], related to severe vasculitis often associated with large areas of necrosis and haemorrhages, may not exclude a potential role of *L. innocua* in exacerbating the lesions found in ID 21.

Serological analysis in 41% of the animals succeeded in detecting morbillivirus infection in 38% (ID 6, 11, 14, 17, 28). The negative serological findings of DMV-positive cases (ID 1, 3, 15, 16, 30) by PCR, given the simultaneous detection of other abs (anti-*T. gondii*), supports the false-negative results, which are reported to occur in serological tests performed with heterologous *Morbillivirus* strains [32]. The absence of abs in 23% of the animals (ID 4, 18, 23) could indicate a possible immunocompromised host response or false-negative results, as reported before.

There is abundant literature on demyelinating lesions in morbillivirus infection in pinnipeds and dogs [13,14,15,16], probably in relation to the well-documented high phylogenetic correlation between CDV and phocine distemper virus [16]; however, few descriptions of myelin changes have been reported in cetaceans with CeMV infection so far [12,17,18,19]. In some cases confirmed by Luxol Fast Blue staining, the procedure has not always proven effective in showing demyelination [20].

Here we provide a comprehensive description of neuropathological changes in the cerebral and the cerebellar cortex of three animals characterized by severe spongiosis by means of double IF staining with specific biomarkers targeting the myelin and the brain cell populations (neurons and glial cells) coupled with the anti-CDV ab. The analysis with anti-myelin marker detected severe myelinopathy, with a reduction in myelin density and large areas completely devoid of myelin, confirming demyelination in the animals. In addition, the anti-CDV marker revealed this alteration in the presence and the absence of direct morbillivirus infection in these brain areas. A plausible explanation for the difference is the different stages of infection since demyelination is associated with viral replication in earlier stages of infection [13].

Double IF analysis using anti-GFAP, anti-NeuN, anti-Iba1 abs and coupled with anti-CDV ab showed cellular activation and viral localization of the brain cell populations inside and around the areas affected by myelinopathy. These findings provide new information about the pathogenesis of the infection.

Unlike previous descriptions of demyelinated areas in CDV infection [13], and despite marked astrocytes activation, we found very few astroglial cells infected with the virus. This observation is shared by an ultrastructural description by means of double IF analysis carried out in cetaceans affected by brain only forms of infection (BOFDI) [61]. We noted marked microglial activation, which was evident in all animals with severe viral infection of the cells. This feature is very common in canine brains with CDV infection, in which oligodendrocyte/myelin damage results from virus-induced microglia activation, which releases myelinotoxic reactive oxygen species [13,14,62].

Dual analysis with anti-NeuN and anti-CDV abs confirmed the neuronal origin of the antigen-bearing cells, as largely documented in CeMV infections [1,20,30,32].

Unfortunately, we obtained no information on oligodendrocytes infection or on oligodendrocytes ultrastructural changes as both anti-Olig2 abs failed to target the oligoglial cells. Therefore, several questions regarding the pathogenesis of demyelination remain unanswered: is the white matter damage a result of microglial activation induced by the virus, which we successfully documented, or rather a consequence of direct viral infection of this cell population, evaluable by observation of co-localization, as previously reported in CDV infection [13,14,62].

Future studies should be carried out using different oligodendrocyte markers, such as 2’–3’–cyclic nucleotide–3’–phosphatase (CNP) or galactocerebroside (GC), associated with the CeMV marker in dual IF. Another future area of focus is the comparative evaluation of anatomical sections of the entire brain and cerebellum to characterize the variety of cell changes occurring during CeMV brain infection.

To the best of our knowledge, this is the first study to describe myelin damage and microglial changes, as evidenced by IF analysis in cetacean brains affected by CeMV infection. The study provides novel data on ultrastructural pathology and neuropathogenetic alteration in systemic CeMV infection. To date, only one study on BOFDI forms has been published [61]. Finally, this is the first study to use confocal laser-scanning microscopy for in-depth analysis of CeMV infection.

## 5. Conclusions

A novel DMV strain, named NE-Atlantic strain, started to replace the DMV Mediterranean strain circulating in Italian waters by late 2015. Based on molecular and immunohistochemical data, this new strain seems to possess a higher neurotropic potential and to cause more severe acute/subacute disease than previously observed during the 2011 and 2013 UMEs. The most frequently observed DMV-associated lesions were astro-microgliosis, neuronal necrosis, spongiosis, malacia, and non-suppurative meningoencephalitis. Co-infection or secondary infection in one-third of the animals may have been a contributory factor to stranding. A myelin-specific biomarker proved effective in demonstrating myelin damage in cetaceans with DMV infection. Morbillivirus antigen-bearing cells of neuronal and microglia origin inside and around demyelinated areas were associated with marked astro and microglia reactivity. Very little morbillivirus staining was evident in astrocytes. These findings further our understanding of DMV-associated brain lesions, shed light on their pathogenesis, and underscore the importance of CNS examination during forensic analysis.

## Figures and Tables

**Table 1:** Primary antibodies for neuropathological characterization in demyelinated/hypomyelinated areas by means of double IF and double IHC.

Antigen	Target	Antibody/Antiserum	Host	Dilution	Source	Technique(s)
CDV-NP	Infected cells	Mono	Mouse	1:500	VMRD	IF, IHC
GFAP	Astrocyte	Poly	Rabbit	1:1000	Millipore	IF
Iba-1	Microglia	Poly	Rabbit	1:1000	Wako	IF
Myelin-PLP	Myelin	Poly	Rabbit	1:500	Abcam	IF
NeuN	Neuron	Poly	Rabbit	1:1000	Abcam	IF
Olig2	Oligodendrocyte	Poly	Rabbit	1:250	Millipore	IF, IHC
Olig2	Oligodendrocyte	Mono	Rabbit	1:100	Abcam	IHC

Legend: CDV denotes canine distemper virus; NP nucleoprotein; PLP proteolipid protein; poly polyclonal; mono monoclonal; IF immunofluorescence; IHC immunohistochemistry.

**Table 2:** DMV-associated lesions in CNS, stage of infection, and co-infection in tissue samples from 31 animals.

Case No.	Cerebral and Cerebellar Cortex								Lesions in Other Regions	Associated Lesions	SI	Co-Infections	References
	M	PC	Astro-Mg	Malacia	NN	S	INCIBs	H					
1	++	++	+	-	+++	+	-	+	Diffuse and mild NS meningoencephalitis associated with focal and minimal NS plexus choroiditis	Protozoan tissue cysts and syncytia	S	*T. gondii*	[1,9,10,30,37]
2	+++	+++	+++	+	+++	++	-	+	-	Syncytia	S	-	[1,10]
3	-	+	-	-	-	+++	-	-	Mild NS myelitis	-	A	-	[1,37]
4	-	-	-	-	++	+++	-	+	Multifocal and mild NS plexus choroiditis		A	-	[1]
5	-	+	-	-	-	++	-	-	-	-	A	*T. gondii*	[1]
6	+	++	++++	-	+++	++	-	+	Diffuse and mild NS meningoencephalitis	-	S	-	[10]
7	-	-	+++	++++	++	+++	-	+	-	Syncytia	A	-	
8	-	-	+++	++++	++	+++	-	-	Perivasal oedema	Syncytia	A	-	
9	+	-	+	-	++	++++	-	-	-	-	A	-	
10	-	-	++	++	++++	++++	-	+	-	Purkinje cell loss	A	*L. ivanovii*	[10,12]
11	-	-	++	-	-	++	-	-	Mild NS plexus choroiditis	-	A	*ˠHV* and *Photobacterium damselae* subsp. damselae	[10,12]
12	+	++	++	++	++++	+++	-	+	-	Suppurative encephalitis characterized by degenerate neutrophils	S	-	[10,12]
13	-	-	-	++	+	+++	-	+	-	-	A	-	[10,12]
14	+++	+++	+++	++	++	+	-	+	Diffuse and moderate NS meningoencephalitis associated with focal and minimal NS plexus choroiditis	Protozoan tissue cysts and syncytia Granulomatous encephalitis	S	*Brucella ceti, T. gondii and Photobacterium damselae subsp. damselae*	[28,29]
15	-	-	+	+	-	++	-	-	-	-	A	-	[10]
16	++	+++	+++	++	++	++	-	+	Diffuse and moderate NS meningoencephalitis and oedema	Protozoan tissue cysts and syncytia Granulomatous necrotizing encephalitis	S	*T. gondii* and *Salmonella* 1,4,[5],12:i:-; .	[10,27]
17	+++	++	-	-	-	++	-	+	Diffuse and moderate NS meningoencephalitis and myelitis	-	C	-	
18	++	-	+	-	++	+++	-	+	-	-	S	-	
19	++++	++++	++++	-	+++	+++	+	+	Multifocal and mild NS plexus choroiditis	Vasculitis, pyogranulomatous encephalitis and syncytia	S	-	
20	++	+	++	++	-	+	-	-	-	Perivasal oedema	S	*ˠHV+* and *T. gondii*	
21	++	++	++	+++	+++	-	-	+	Diffuse and mild NS meningoencephalitis	Vasculitis, syncytia	S	*Photobacterium damselae* subsp. *damselae*, *Listeria innocua*	
22	++	++	+++	-	+++	++	-	-	Oedema	-	S	-	
23	++	+++	+++	+++	++++	+++	-	-	Diffuse and moderate NS meningoencephalitis	Syncytia	S	-	
24	++	++	+++	-	+++	++	-	+	Focal minimal NS plexus choroiditis	Protozoan tissue cysts, syncytia, and Purkenje cell loss; granulomatous encephalitis	S	*T. gondii*	
25	-	-	-	-	-	+++	-	-	-	-	A	-	
26	+++	++	-	-	-	++	-	-	-	Protozoan tissue cysts; granulomatous encephalitis	S	*T. gondii*	
27	-	-	+++	-	++	-	-	-	-	Syncytia	A	-	
28	++	+++	+++	-	+++	++++	-	-	Diffuse and moderate NS meningoencephalitis	-	S	-	
29	+++	+++	+++	++	++	-	-	-	-	-	S	*T. gondii*	
30	-	-	++	-	+	+++	-	-	-	-	A	*T. gondii* and *Photobacterium damselae subsp. damselae*	
31	++	+++	+++	+	+++	-	-	+	-		S	*Photobacterium damselae* subsp. *damselae*	

Legend: DMV, dolphin morbillivirus; CNS, central nervous system; NS, non suppurative; M, meningitis; PC, perivascular cuffing; Mg, microgliosis; INCIBs, intranuclear and/or intracytoplasmatic inclusion bodies; NN, neuronal necrosis; H, haemorrhage; S, spongiosis. Absent (-); minimal (+); mild (++); moderate (+++); severe (++++); SI, stage of infection; A, acute; S, subacute; C, chronic.

## Data Availability

The data presented in this study are available within the article and the Supplementary Materials.

## Supplementary Materials

The following are available online at https://www.mdpi.com/2076-2615/12/4/452/s1, Figure S1: Western Blot to evaluate specific reactivity of different polyclonal anti- bodies against Stenella coeruleoalba; Table S1: Anamnestic and stranding data of thirty-one animals under study; Table S2: Results of molecular, immunohistochemical, microbiological and serological investigations and type of infection of thirty-one animals under study.
