# Supplementary material for: Neuropathological Characterization of Dolphin Morbillivirus Infection in Cetaceans Stranded in Italy
Source: Animals (Basel). 2022 Feb 12;12(4):452. doi: 10.3390/ani12040452 (PMC8868427; doi:10.3390/ani12040452)
Supplement: Supplementary file 1 [file animals-12-00452-s001.zip › animals-1556833.pdf]

animals

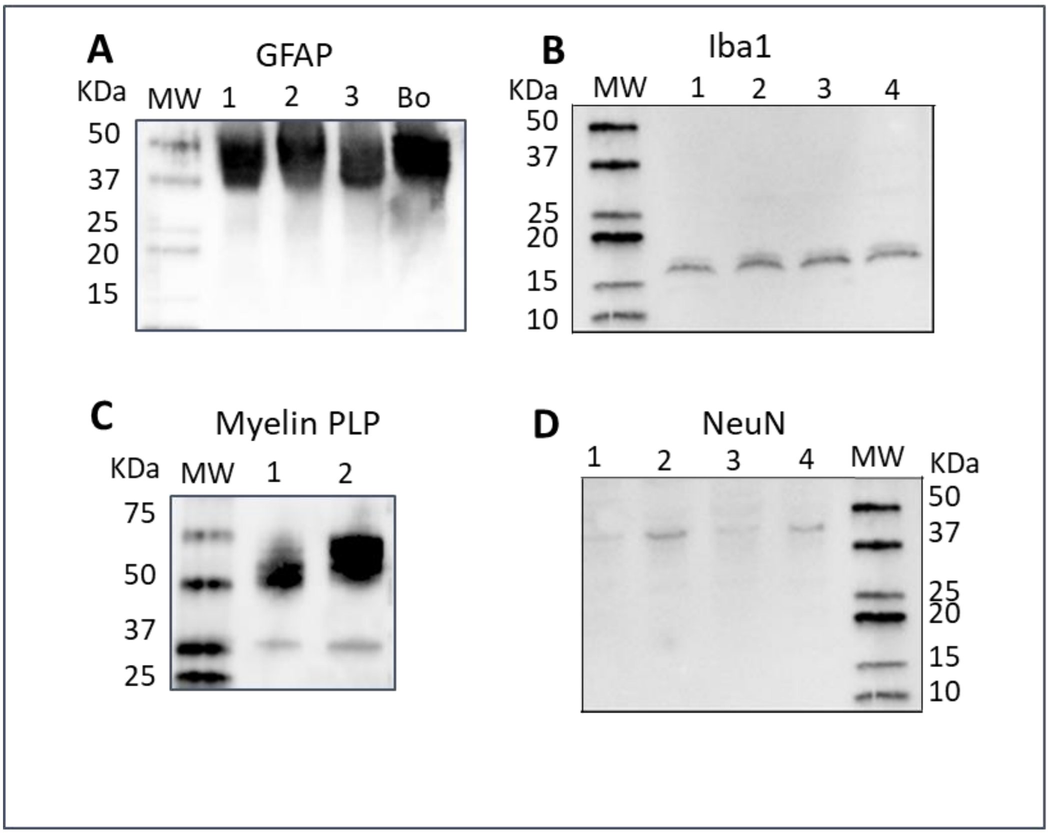

Supplementary material Figure S1. Figure S1. Western Blot to evaluate specific reactivity of different polyclonal antibodies against *Stenella coeruleoalba*.

Central nervous system (CNS) specimens from *Stenella coeruleoalba* (lanes 1, 2, 3 and 4) or *Bos taurus* (lane Bo) were homogenized at 10% weight/volume and subjected to immunoblotting. Twenty to forty µg of total protein for each sample were reduced, loaded on Mini-PROTEAN TGX 4-20% Gels (#456-1094, Biorad) and separated electrophoretically in XT MES running Buffer 1X (Biorad) for about 50 minutes at 150V using the MiniPROTEANII electrophoretic chamber (Biorad). The proteins transfer was obtained with a voltage of 25 V for 5 minutes using the semi-dry Trans-Blot-Turbo (Biorad) transfer system according to the manufacturer's protocols. Detection of proteins was carried out respectively with: **A)** anti-cow GFAP polyclonal antibody (1:10000 over night, DAKO#Z0334, predicted molecular weight ≈ 50 Kda); **B)** anti-Iba1 polyclonal antibody (1:2500 over night, Biocare Med.#CP290, predicted molecular weight ≈ 16.7 Kda; **C)** anti-myelin PLP polyclonal antibody (1:2500 over night, abcam#ab28486, predicted molecular weight ≈ 35 Kda + isoforms/duplicates from literature); **D)** anti-NeuN polyclonal antibody (1:2500 over night, Abcam#ab104225, predicted molecular weight ≈ 48 KDa).

Membranes were incubated with a 1:5000 dilution of the HRP-Goat anti-Rabbit antibody (Invitrogen # 656120) and with 1 ml of the combined 1:1 solution of the Clarity™ Western ECL Substrate detection kit (Biorad). Finally, membranes were acquired in chemiluminescence using the ChemiDoc™ Touch (Biorad), image acquisition system or equivalent system. MW, molecular size markers (in kilodaltons). The data are representative of multiple immunoblots of these specimens.

Supplementary material Table S1. Anamnestic and stranding data of thirty-one animals under study

ID Case	Ref. Lab	Species	DCC	NS	Sex	Age	SL	SD
1	123517/08	*Stenella coeluleoalba*	2	Poor	M	Adult	Laigueglia (SV)	24/10/2008
2	80729/2/11	*Stenella coeluleoalba*	2	good	M	Juvenile-Subadult	Eboli (SA)	16/07/2011
3	10331/12	*Stenella coeluleoalba*	3	Good	M	Juvenile-Subadult	Ospedaletti (IM)	24/01/2012
4	42303/13	*Stenella coeluleoalba*	3	Poor	M	Juvenile-Subadult	S. Lucido (CS)	03/04/2013
5	71891/14	*Stenella coeluleoalba*	3	Good	M	Juvenile-Subadult	S.Giovanni a Piro (SA)	29/07/2014
6	3908/15	*Stenella coeluleoalba*	2	Good	F	Adult	Imperia	16/01/2015
7	80677/16	*Stenella coeruleoalba*	2	Poor	F	Juvenile-Subadult	Eraclea Minoa (AG)	21/08/2016
8	10475/17	*Stenella coeruleoalba*	3	Good	F	Juvenile-Subadult	Riposto (CT)	19/09/2016
9	10477/17	*Stenella coeruleoalba*	2	Poor	F	Juvenile-Subadult	Ispica (RG)	30/09/2016
10	92300/16	*Stenella coeruleoalba*	2	Poor	M	Juvenile-Subadult	Porto Cesareo (LE)	07/10/2016
11	95842/16	*Stenella coeruleoalba*	2	Poor	M	Juvenile-Subadult	Genova	20/11/2016
12	6023/17	*Stenella coeruleoalba*	2	Poor	M	Adult	Otranto (LE)	29/11/2016
13	6020/17	*Stenella coeruleoalba*	2	Poor	M	Juvenile-Subadult	Torricella (TA)	21/12/2016
14	16769/17	*Stenella coeruleoalba*	2	Moderate	M	Juvenile-Subadult	Savona	16/02/2017
15	26823/17	*Stenella coeruleoalba*	3	Moderate	F	Juvenile-Subadult	Prino (IM)	09/03/2017
16	78983/17	*Stenella coeruleoalba*	2	Poor	F	Adult	Savona	14/09/2017
17	106163/17	*Stenella coeruleoalba*	2	Poor	M	Adult	Alassio (SV)	01/12/2017
18	2926/18	*Stenella coeruleoalba*	2	Moderate	M	Juvenile-Subadult	Imperia	13/01/2018
19	41779/18	*Stenella coeruleoalba*	1	Poor	M	Adult	Schiavonea (CZ)	18/04/2018
20	62728/18	*Stenella coeruleoalba*	2	Poor	M	Adult	Bivona (VV)	27/06/2018

ID Case	Ref. Lab	Species	DCC	NS	Sex	Age	SL	SD
21	87558/18	*Stenella coeruleoalba*	2	Moderate	F	Juvenile-Subadult	Arenzano (GE)	29/10/2018
22	100927/18	*Stenella coeruleoalba*	1	Good	M	Adult	Centofontane (CZ)	19/11/2018
23	619/19	*Stenella coeruleoalba*	2	Poor	M	Adult	Alassio (SV)	03/01/2019
24	9553/19	*Stenella coeruleoalba*	1	Good	F	Juvenile-Subadult	Nicotera (VV)	09/01/2019
25	23352/19	*Stenella coeruleoalba*	2	Poor	F	Adult	Ispani (SA)	27/01/2019
26	25089/19	*Stenella coeruleoalba*	2	Moderate	M	Juvenile-Subadult	Palmi (RC)	29/01/2019
27	23336/19	*Stenella coeruleoalba*	3	Poor	M	Adult	Torre del Greco (NA)	08/02/2019
28	21724/19	*Stenella coeruleoalba*	2	Good	F	Adult	Savona	05/03/2019
29	12060/20	*Stenella coeruleoalba*	3	Good	F	Adult	Mondragone (CE)	03/04/2019
30	59260/19	*Tursiops truncatus*	2	Moderate	M	Adult	Genova	04/07/2019
31	38375/20	*Tursiops truncatus*	3	Good	M	Juvenile-Subadult	Parghelia (VV)	22/04/2020

Legend: ID: identification; Ref. lab: Laboratorial reference; DCC: decomposition conservation code; NS: nutritional status; F: female; M: male; SL: stranding location; SD: stranding date.

animals

MDPI

Supplementary material Table S2. Results of molecular, immunohistochemical, microbiological and serological investigations and type of infection of thirty-one animals under study

ID case	DMV strain	Disease type	PCR			IHC		Bacteria	Sierology			References
			T. gondii	HV	*Brucella* spp.	CDV	*T. gondii*		CDV	*T. gondii*	*Brucella* spp.	
1	Med	Systemic	+	-	-	+	+	negative	-	1:80 (serum)	-	[1,9,10,30,37]
2	Med	NA	-	-	-	+		negative	NP	NP	NP	[1,10]
3	Med	Systemic	-	-	-	-		negative	-	>1:160	-	[1,37]
4	Med	Systemic	-	-	-	-		negative	-	-	-	[1]
5	Med	Systemic	+	-	-	+	-	negative	NP	NP	NP	[1]
6	Med	Systemic	-	-	-	-		negative	1:40 (serum)	1:80 (serum)	-	[10]
7	NE-atlantic	Systemic	-	-	-	+		negative	NP	NP	NP	
8	NE-atlantic	Systemic	-	-	-	+		negative	NP	NP	NP	
9	NE-atlantic	Systemic	-	-	-	+		negative	NP	NP	NP	

**ID case**	**DMV strain**	**Disease type**	**PCR**			**IHC**		**Bacteria**	**Sierology**			**References**
			T. gondii	**HV**	***Brucella* spp.**	**CDV**	***T. gondii***		**CDV**	***T. gondii***	***Brucella* spp.**	
10	NE-atlantic	Systemic	-	-	-	+		*L. ivanovii*	NP	NP	NP	[10,12]
11	NE-atlantic	Systemic	-	ᵞHV	-	+		*Photobacterium damselae*	1:40 (serum)	>1:40 (serum)	-	[10,12]
12	NE-atlantic	Systemic	-	-	-	+		negative	NP	NP	NP	[10,12]
13	NE-atlantic	Systemic	-	-	-	-		negative	NP	NP	NP	[10,12]
14	NE-atlantic	Systemic	+	-	+	+	+	*Brucella ceti Photobacterium damselae*	1:8 (serum)	>1:40 (serum, CSF and aqueous hu-mor)	-	[28,29]
15	NE-atlantic	Systemic	-	-	-	+		negative	-	>1:40 (serum)	-	[10]
16	NE-atlantic	Systemic	+	-	-	+	+	*Salmonella* 1,4,[5],12:i:-; .	-	IFAT >1:640 (serum and aqueous hu-mor)	-	[10,27]
17	NE-atlantic	Systemic	-	-	-	-		negative	>1: 256 (serum)	-	-	
18	NE-atlantic	Systemic	-	-	-	-		negative	-	-	-	
19	NE-atlantic	Systemic	-	-	-	+		negative	NP	NP	NP	
20		Systemic	+	ᵞHV	-	+	+	negative	NP	NP	NP	

ID case	DMV strain	Disease type	PCR			IHC		Bacteria	Sierology			References
			T. gondii	HV	*Brucella* spp.	CDV	*T. gondii*		CDV	*T. gondii*	*Brucella* spp.	
21	NE-atlantic	Systemic	-	-	-	+		*Photobacterium damselae, Listeria innocua*	NA	NA	NA	
22		Systemic	-	-	-	+		negative	NP	NP	NP	
23	NE-atlantic	Systemic	-	-	-	+		negative	-	-	-	
24	NE-atlantic	Systemic	+	-	-	+	+	negative	NP	NP	NP	
25	NE-atlantic	Systemic	-	-	-	-		negative	NP	NP	NP	
26	NE-atlantic	Systemic	+	-	-	+	+	negative	NP	NP	NP	
27	NE-atlantic	Systemic	-	-	-	+		negative	NP	NP	NP	
28	NE-atlantic	Systemic	-	-	-	+		negative	1:128 (humor aqueous)	-	-	
29	NE-atlantic	Systemic	+	-	-	+	-	negative	NP	NP	NP	
30	NE-atlantic	Systemic	+	-	-	+	-	*Photobacterium damselae*	-	>1:40 (serum and aqueous humor)	-	
31	NE-atlantic	Systemic	-	-	-	+		*Photobacterium damselae*	NP	NP	NP	

Legend: ID: identification; PCR: polymerase chain reaction; HV: Herpesvirus; IHC: immunohistochemistry; CDV: canine distemper virus; NE-Atlantic: Northeast Atlantic; Med: Mediterranean.

[1]: 10.7589/2017-02-035
[9]: 10.1038/s41598-018-19269-2
[10]: 10.1038/s41598-020-77835-z
[12]: 10.3354/dao03323
[27]: 10.1038/s41598-019-42474-6
[28]: 10.1371/journal.pone.0240178
[29]: 10.3390/ani11051201

[30]: 10.1556/004.2021.00028
[37]: 10.7589/2016-07-150
